# Development of a multidisciplinary cooperative nutrition management process for critically ill patients—a Delphi expert consensus study

**DOI:** 10.3389/fnut.2026.1793038

**Published:** 2026-06-19

**Authors:** Shunxia Sun, Jin Yang, Juan Huang, Jiangqiong Peng, Xiaoling Tang

**Affiliations:** Chongqing General Hospital, Chongqing, China

**Keywords:** critically ill patients, expert consensus, management protocol, multidisciplinary team, nutrition management

## Abstract

**Objective:**

To explore a multidisciplinary cooperative nutrition management process for critically ill ICU patients in China for providing a standardized nutrition management process and improving the effect and safety of nutritional support.

**Methods:**

Our first draft of the “multidisciplinary nutrition management process for critical patients” and “enteral nutrition management process for critical patients”, which included five steps and 31 criteria, was prepared through literature review and analysis, referring to the domestic and foreign nutrition management guidelines for critical patients, and referring to the nutrition management process for critically ill patients abroad. We recruited consulting experts from Beijing, Zhejiang, Jilin, Guangdong, and Chongqing in China. Using the Delphi method, we conducted expert consultations to evaluate the initially prepared standardized process, subsequently refining and adjusting items based on their feedback.

**Results:**

After two rounds of expert consultation, all 20 invited experts completed both rounds, 15 experts provided written opinions, and 43 expert opinions were collected. The nutrition management process was bifurcated into “multidisciplinary nutrition management process for critically ill patients” and “enteral nutrition management process for critically ill patients,” which collectively included five steps (within 24 h of admission, within 24–48 h of admission, after 48 h of admission and during hospitalization, during transfer to another department, and during discharge) and 31 standard processes. The 31 criteria were rated as desirable or necessary by 17–20 evaluators (85–100%).

**Conclusion:**

The multidisciplinary cooperative nutrition management process for critically ill patients established by us using the Delphi method is scientific and standardized and shows promise as a practical tool that may improve for nutritional management.

**Relevance to clinical practice:**

Structured multidisciplinary nutrition management operational processes can guide clinical practice. They has potential for implementation in similar settings in the clinical nutrition management of critically ill patients in the ICU or other departments.

## Introduction

Nutritional support therapy is essential for critically ill patients (1). The prevalence of malnutrition is alarmingly high in the ICU, with recent global initiatives indicating that up to 78% of critically ill patients are at high nutritional risk at the time of admission or develop malnutrition during their hospitalization due to catabolic stress. Elevated nutritional risk is independently associated with adverse clinical outcomes, including increased mortality, prolonged mechanical ventilation, and persistent long-term functional disability and cognitive impairment in survivors (2, 3). Critically ill patients often present with multiple comorbidities and complex clinical profiles, placing them at a significantly higher risk for nutritional deficiencies. At the same time, nutrition treatment for critically ill patients requires a multidisciplinary approach, and the relatively basic nutrition management from the ICU medical staff is inadequate to meet the nutrition requirements of the patient (4).

A clinical multidisciplinary working team ideally involves teams from three or more related disciplines that conduct regular clinical seminars on diseases or clinical concerns associated with their specialization; this collaboration of multiple disciplines is aimed to formulate a personalized diagnosis and treatment plan for patients (5). A multidisciplinary team can avoid the diagnostic bias of a single discipline (1, 4), reasonably and effectively use and combine medical resources, and provide patients with more professional, scientific, and reasonably optimized treatment and nursing programs. Empirical evidence strongly supports the efficacy of the multidisciplinary team model. Large-scale cohort studies have reported an independent association between the implementation of specialized nutrition support teams (NSTs) and significant reductions in in-hospital mortality (up to 40% in specific cohorts). Furthermore, systematic reviews indicate that NSTs effectively reduce preventable complications like catheter-related infections and metabolic derangements, thus enhancing both patient safety and economic efficiency (6–9).

In recent years, the multidisciplinary team cooperative model has become an increasingly important part of modern medicine and has been implemented in various medical fields throughout the treatment process. Its use has been reported in determining patient treatment plans, hospital infection control, trauma first aid, patient rehabilitation, medical education, and translational medicine (8–13). Research related to the nursing specialty mainly focuses on the continuous nursing of patients with chronic diseases, health education, perioperative nursing, and hospice care (12, 14–16). Increasing multidisciplinary team cooperation in clinical practice has laid a good foundation for the expansion of its use in several medical fields (15, 17, 18).

Nutritional support treatment for critically ill patients covers the basic nutritional and metabolic needs of patients while considering the nutrient composition of food and patients’ digestive and absorption ability (19). Nutritional support treatment is crucial for critically ill patients (20, 21). Nutritional support treatment involves many fields, such as clinical diagnostics, pharmaceutics, nutrition, and nutritional support nursing (22). Relying solely on the ICU medical staff to formulate and provide optimal nutritional treatment schemes and take appropriate measures is not advisable. In European and American countries, nutritional treatment schemes for critically ill patients are typically jointly formulated by ICU doctors, specialists, nutritionists, and clinical pharmacists (20, 23–26), ensuring reasonable nutrition supply and disease recovery (8). However, the multidisciplinary team cooperation model had a late start in China. Nutrition management of critically ill patients is currently not comprehensive enough to achieve multidisciplinary team cooperation (11), with literature analysis identifying few reports or guidelines on multidisciplinary team cooperation in the nutrition management of critically ill patients in China (17).

Consensus group methodologies, such as the Delphi method, are used to systematically synthesize expert opinions when evidence is lacking or when experimental and epidemiological methods do not yield desired outcomes (25, 27). The construction of a multidisciplinary collaborative nutrition management process for critically ill patients needs input from experienced clinical experts to ensure the operability and adaptability of the process (13, 15, 17).

Based on the guidelines for the nutrition treatment of critically ill patients abroad and referring to nutrition management schemes for critically ill patients implemented overseas, this study intends to (i) build a preliminarily multidisciplinary team cooperative nutrition management model and process for critically ill patients in China and (ii) provide a theoretical reference for implementing multidisciplinary nutrition management of critically ill patients in China.

## Participants and methods

### Design

The multidisciplinary nutrition management model for critically ill patients was established using the Delphi expert consultation method, which has strong applicability and operability and can collate expert opinions from different regions (25, 27). According to the Delphi method during January 2023, expert opinions were collected through two rounds of consultation, which could have been extended to a third round if necessary. The research team summarized the best evidence and referred to the preliminary nutrition management model items of the international multidisciplinary nutrition management models for critically ill patients. During both rounds of consultation, the scores of experts on the importance of each item in the questionnaire were anonymously collected, and anonymized inputs from experts guided modifications in the second round. The second round of expert consultation was conducted after a summary was drawn from the first round of responses.

### Participants

The project manager used the purposive sampling method to select the consulting experts for this study from across the country. Purposive sampling was used via professional networks.

We included experts who (i) had senior professional titles, (ii) more than 10 years of treatment and nursing experience in critically ill patients, (iii) were familiar with the knowledge of nutrition management of critically ill patients and had extensive management experience, and (iv) were interested and willing to participate in this study. We excluded experts who (i) were engaged in other work in the ICU and had never interacted with the patient’s nutrition manager or (ii) had relevant work experience but had not been involved in clinical work for 10 years or longer. The consulting experts mainly evaluated the applicability of items and provided their own suggestions and opinions (28).

### Steering group

A steering group was created to supervise the research project, make strategic decisions on research design-related aspects, and collect data and perform relevant analyses, and the members had to reach a consensus on the final steps and criteria before implementation. The group also generated a set of initial criteria for the Delphi process and managed survey administration. The group comprised one ICU chief physician (JY), two deputy chief physicians (YL and JJ), one deputy chief nurse (XT), two chief nurses (JH and JP) and one clinical nutritionist (QW). To avoid the contamination or repetition of Delphi results, the members of the steering group were not among the expert consulted (29).

### Survey development

Through literature review and analysis, the members of the steering group generated a summary of evidence on the nutrition management of critically ill patients. Furthermore, incorporating the processes and steps of multidisciplinary nutrition management models used for critically ill patients abroad, they formed the first draft of the multidisciplinary enteral nutrition management process for critically ill patients in China, which was bifurcated into “multidisciplinary nutrition management process for critically ill patients” and “enteral nutrition support process for critically ill patients.” The “enteral nutrition support process” is a subprocess of the “multidisciplinary nutrition management process for critically ill patients.” The “multidisciplinary nutrition management process for critically ill patients” includes 16 criteria and focuses on patient nutrition at five specific time points: admission, within 24 h of admission, within 24–48 h of admission, during hospitalization, and transfer and discharge. The “enteral nutrition support process” includes 15 criteria. For seamless communication, the steering group interacted on a WeChat group, where the preliminary drafts of the process standards were shared as Tencent online documents for each member to review and modify. All items were fully reviewed and discussed by the group members. Once no further modifications were required, the first round of the Delphi expert consultation questionnaire, which had two parts, five steps, and 31 criteria, was commenced (Table 1).

**Table 1:** Content of the Delphi expert consultation questionnaire on the “multidisciplinary nutrition management process for critically ill patients”.

Part	Step	Criteria
Multidisciplinary nutrition management process for critically ill patients	Admission	Nutrition risk screening
	Within 24 h of admission	ICU doctors use the NUTRIC score table to complete the nutritional risk screening, and the nurses measure the patient’s height, weight
		Is the score ≥ 5 or not?
		The ICU doctor in charge evaluates the score and determines the nutritional support route
		The ICU doctor in charge ensures that nutrition risk screening is performed on a weekly basis
	Within 24–48 h of admission	A dietitian is consulted for assessment of nutritional requirements
		The need for enteral nutrition is determined
		Physical measurement indicators, dietary intake, gastrointestinal symptoms, and laboratory indicators are assessed
		The need for enteral nutrition is re-evaluated
		ICU doctors prescribe enteral nutrition according to the “flow chart of enteral nutrition support for critical patients,” and ICU nurses cooperate to complete the establishment of nutrition channels. A radiologist confirms the positioning of enteral nutrition devices
		Parenteral nutrition support
		A clinical nutritionist suggests the parenteral nutrition scheme, and the doctor gives nutritional advice A clinical pharmacist reviews the orders and provides recommendations for optimization A nurse places a PICC or a doctor places a CVC catheter. A radiologist confirms the positioning of these devices
	During hospitalization	ICU nurses implement the nutritional support and monitor the effect of nutritional support
		ICU nurses administer enteral or parenteral nutrition support according to the doctor’s advice ICU nurses use ultrasound to monitor the amount of gastric retention ICU nurses ensure the completion and observe the effects of nutritional support The ICU doctor in charge re-evaluates the NUTRIC score every week, adjusting the nutritional support route and dose according to the changes in the patient’s condition and ensuring the completion of nutritional support. The clinical dietitian is consulted if necessary
	Transfer and discharge	The nurses collect relevant data
		ICU nurses collect relevant data of patients, such as general data, mechanical ventilation days, ICU hospitalization days, total hospitalization days, total hospitalization expenses, antibiotic expenses, and disease outcome When the patient is being transferred to another department or discharged from the hospital, the ICU nurse again uses the NUTRIC scoring table to evaluate the nutritional risk and completes shift handover to the new department according to the screening results; nurses give relevant diet-related health education to family members of the discharged patient
Enteral nutrition support process for critically ill patients		The ability of the patient to eat by mouth is evaluated
		Oral feeding is commenced if the need for enteral nutrition is ruled out
		Whether the patient has any contraindications to enteral nutrition is evaluated Absolute contraindications are as follows: Mechanical intestinal obstruction Intestinal ischemia Relative contraindications are as follows (individualized assessment required): Hemodynamic instability Short bowel syndrome Intestinal anastomosis High-output intestinal fistula > 1,500 mL/d
		Whether the patient has any contraindications to intragastric feeding is evaluated, which were indicated by the following: The gastric residual volume remaining under 250 mL despite the use of gastric motility-promoting drugs The presence of chronic/acute gastroesophageal reflux High risk of lung aspiration despite preventive measures
		Consider parenteral nutrition (PN)/enter PN process
		The feasibility of duodenal/jejunal nutrition is evaluated, which is given as follows (if required): - Intragastric nutrition - Short term < 4 to 6 weeks - Long term > 4 to 6 weeks
		An ICU nurse places a naso-intestinal tube under ultrasound guidance; the radiologist confirms the positioning The doctors/nurses in the ICU can place a naso-intestinal tube under endoscopic guidance
		The ICU doctor in charge places a percutaneous endoscopic jejunostomy (PEJ) tube, or a surgeon performs an enterostomy
		An ICU nurse places a gastric tube
		The ICU doctor in charge places a PEJ tube, or a surgeon performs a gastrostomy
		ICU doctors discuss nutrition plans with clinical nutritionists to determine the appropriate enteral formula: patients with diabetes receive Ruidai, those with high calorie demand but limited fluid intake receive Ruixian, and those with low immunity receive Jiaweiti or other nutrition preparations
		Enteral nutrition therapy in the ICU: The initial speed is 20 mL/h, which is increased by 10 mL/h every 4–6 h until the target dose is reached. The target dose is determined by ICU doctors

### The Delphi method

#### Round 1

The selected experts were contacted in advance, and the investigation was initiated after obtaining informed consent from the consulting experts. The first round of the questionnaire included three parts: a letter to the experts, the main body of the questionnaire, and basic information about the consulting experts. The purpose and significance of the study and the questionnaire fields to be filled by the experts were introduced in the letter. In the second part—the main body of the questionnaire—the experts scored the applicability of all items according to Likert’s 3-level scoring method (applicable, general, and not applicable) and provided opinions or suggestions on the steps and criteria of the standard processes (e.g., time period division, text description, grouping, and sequence). The consulting experts were not required to provide qualitative opinions or suggestions; however, they had to select the adaptive Likert level for each item and could choose to provide other feedback. The third part was to collect the basic information of experts, including their name, gender, age, work organization, professional title, position, and educational background. They were also required to mandatorily fill in the familiarity with the survey and provide the reasoning behind their judgments. The questionnaires were administered *via* email. The consulting experts could stop responding to the questionnaire at any time during the consultation, and the questionnaire had to be responded within 2 weeks.

#### Round 2

The steps and standards of the process were adjusted on the basis of the results of the first round of expert consultation. The standards that met the inclusion criteria were retained, and those meeting the exclusion criteria were discussed in a group and then eliminated if a consensus was reached. The standards were revised based on the specific modification suggestions provided by the consulting experts. The experts were shown a summary of everyone else’s opinion before they were asked the same questions again in the second round, again via e-mail. As they did in the first round, the experts used Likert’s 3-level scoring method to score the applicability of all items and provide modification suggestions on the standard time period division, text description, grouping, sequence, and other aspects. The experts were asked to provide structured feedback within 1 week, which included both quantitative applicability ratings (using the same 3-point Likert scale) and qualitative suggestions for modification. This feedback directly informed the final adjustments to the criteria.

### Data analysis

EpiData 3.1 software (The EpiData Association, Odense, Denmark) was used for dual-entry data input and database establishment. SPSS 21.0 software (IBM Corp., Armonk, NY, USA) was used for statistical analysis. General data of the consulting experts and presurvey objects were statistically described as percentages, means, standard deviations, or other statistical indicators. In the first round of consultation, when the proportion of experts choosing “applicable” or “not applicable” was >80%, we considered that the experts had reached a consensus on retaining or excluding an item. After the first round of expert consultation, a steering group meeting was held to review and discuss the item rating and quality feedback, including suggestions on adjusting the text description, adding items at any level, and general comments or suggestions. If two or more consulting experts provided suggestions or if the steering group members agreed with the suggested text description adjustment, reordering, or merging, the corresponding entries were revised. The results of the second round of expert consultation were then handled according to the same process.

Questionnaires with incomplete answers were excluded. The steering group decided on the need for a third round of consultation on the basis of the results and feedback of the first two rounds of consultation.

#### Ethics

The study was approved by the Ethics Committee of Chongqing General Hospital (Approval No.: 2019ZDXM020) and was conducted in accordance with the ethical principles of the Declaration of Helsinki. All consulting experts provided informed consent and participated voluntarily. This report follows the ACcurate COnsensus Reporting Document (ACCORD) guidelines.

## Results

### Consulting expert information

A total of 30 questionnaires were distributed in the first round of correspondence, and 66.6% (*n* = 20/30; 8 men, 12 women) of consulting experts completed the questionnaire survey. Seven experts dropped out, with five not providing feedback and two submitting partially completed questionnaires. Three experts could not participate because of time constraints. The average age of the 20 consultants was 42.1 ± 6.19 years (range, 29–49 years); their overall work experience was 20.1 ± 9.47 years (range, 3–31 years), with 13.6 ± 7.7 years (range, 2–25 years) of work experience in the ICU. Their geographical distribution was five in Chongqing (25%), four in Beijing (20%), four in Zhejiang (20%), four in Guangdong (20%), and three in Jilin (15%). Among them were 8 doctors (40%), 8 nurses (40%), and 4 nutritionists (20%). See Table 2 for details of the experts.

**Table 2:** General information of the experts in two rounds of correspondence.

Characteristics	Round 1 and 2 (both, *n* = 20)
Gender	
Male	8
Female	12
Occupation	
Doctor	8
Nurse	8
Dietitian	4
Academic title	
Intermediate title	4
Associate senior title/associate professor	10
Senior title/professor	6
Regional distribution	
Chongqing	5
Beijing	4
Zhejiang	4
Jiling	3
Guangdong	4

The 20 experts who completed the first consultation were invited for the second consultation, and all 20 experts completed the second consultation. The information of the experts consulted is consistent between the two rounds.

### Consultation results

The final score given by the consulting experts to each criterion after the first and second consultations is shown in Table 3. Scoring results: In the first consultation, the experts provided 43 general comments. After summarizing the comments, none of the criteria were deleted, added, merged, or subdivided, and 13 criteria were rewritten. In comparison, in the second consultation, the experts provided only three general comments. After summarizing the comments, one previously unmodified indicator was rewritten. Appendix 1 shows detailed expert suggestions. Consultation results were bifurcated into “multidisciplinary cooperative nutrition management process for critically ill patients” and the “enteral nutrition support process,” and the results of both rounds of consultation have been described separately.

**Table 3:** Expert adaptability score of the “multidisciplinary nutrition management process for critically ill patients”.

Part	Step	Criteria	Suitability score (*X* ± *S*)	Proportion of “fit” option (%)
Multidisciplinary nutrition management process for critically ill patients	Admission		3.00 ± 0.00	100.00%
		Nutrition risk screening	2.90 ± 0.10	90.00%
	Within 24 h of admission		2.95 ± 0.07	95.00%
		The ICU doctor completes the nutritional risk screening using the NUTRIC score table, and a nurse collects data on the patient’s height and weight. For obese patients (BMI ≥ 30 kg/m^2^), adjusted body weight (ABW) is recommended for nutritional calculation	2.85 ± 0.13	85.00%
		Whether the score is ≥ 5 is determined	2.85 ± 0.13	85.00%
		The ICU doctor in charge conducts nutritional risk screening once every 2 days	2.85 ± 0.13	85.00%
		The ICU doctor in charge evaluates and determines the nutritional support route	2.90 ± 0.10	90.00%
	Within 24–48 h of admission		2.90 ± 0.10	90.00%
		The patient’s eligibility for enteral nutrition is assessed	2.85 ± 0.13	85.00%
		This assessment is performed by a clinical nutritionist and an ICU physician	2.90 ± 0.10	90.00%
		Assess the physical measurement indicators, dietary intake, gastrointestinal symptoms, and laboratory indicators, among other aspects	2.90 ± 0.10	90.00%
		The patient’s eligibility for enteral nutrition is re-assessed	2.95 ± 0.07	95.00%
		ICU doctors prescribe enteral nutrition according to the “enteral nutrition support process for critically ill patients,” and ICU nurses cooperate with the doctors to complete the establishment of nutrition channels. If a small intestinal feeding tube is installed, the radiologist evaluates the tube position using X-ray	2.95 ± 0.07	95.00%
		Parenteral nutrition support:	3.00 ± 0.00	100.00%
		The clinical nutritionist recommends the ideal parenteral nutrition scheme, and the doctor prescribes the nutritional treatment and sets the nutrition target value, as well as protein and energy targets A clinical pharmacist reviews the orders and provides recommendations for optimization A nurse places a PICC, or the doctor places a CVC, and a radiologist confirms the catheter position	2.95 ± 0.07	95.00%
	48 h after hospitalization		2.90 ± 0.10	90.00%
		ICU nurses initiate nutritional support and monitor its effects	2.90 ± 0.10	90.00%
		An ICU nurse initiates enteral or parenteral nutrition support according to the doctor’s advice ICU doctors initially monitor gastric retention with ultrasound, and then, nurses complete the ultrasonic monitoring on a daily basis ICU doctors and nurses evaluate the completion and effect of nutritional support The ICU doctor in charge re-evaluates the nutritional score every 2–3 days, adjusts the nutritional support route and dose according to the patient’s evolving condition, and eventually takes the patient off nutritional support	2.95 ± 0.07	95.00%
	Transfer and discharge		3.00 ± 0.00	100.00%
		The ICU nurses collate the data	2.95 ± 0.07	95.00%
		ICU nurses assess the nutritional risk using the NUTRIC scale. The NUTRIC score should be interpreted with caution at discharge, as a low score does not rule out ongoing nutritional needs (e.g., due to dysphagia). According to the screening results, ICU nurses complete shift handover to the new department, and nurses give relevant diet-related health education to the family members of the discharged patient	2.95 ± 0.07	95.00%
Enteral nutrition support process for critically ill patients		Whether the patient can eat orally is determined	3.00 ± 0.00	100.00%
		Oral feeding as the mode for nutrition intake	2.90 ± 0.10	90.00%
		Whether the patient has any contraindications to enteral nutrition is determined Absolute contraindications: Mechanical intestinal obstruction Intestinal ischemia or hemorrhage Relative contraindications (individualized assessment required) Hemodynamic instability Short bowel syndrome Intestinal anastomosis A high output intestinal fistula >1,500 mL/L AGI III or IV	2.95 ± 0.07	95.00%
		Any contraindications to intragastric feeding are indicated by the following: The gastric residual volume is still >250 mL despite the use of gastric motility-promoting drugs Chronic/acute gastroesophageal reflux Despite preventive measures, there is still a high risk of aspiration in the lungs	2.95 ± 0.07	95.00%
		Consider parenteral nutrition (PN) /enter PN process	2.90 ± 0.10	90.00%
		The feasibility of duodenal/jejunal nutrition is evaluated	2.95 ± 0.07	95.00%
		Intragastric nutrition - Short term <4 to 6 weeks - Long term ≥4 to 6 weeks	2.95 ± 0.07	95.00%
			2.90 ± 0.10	90.00%
			2.90 ± 0.10	90.00%
		A naso-intestinal tube is placed by various methods, such as under ultrasound guidance by ICU nurses, and the position of the tube is confirmed by a radiologist The ICU physician/nurse places the naso-intestinal tube under endoscopic guidance	2.90 ± 0.10	90.00%
		The ICU doctor in charge places a percutaneous endoscopic jejunostomy (PEJ) tube, or a surgeon performs an enterostomy	2.90 ± 0.10	90.00%
		The ICU nurse places an indwelling gastric tube	2.90 ± 0.10	90.00%
		The ICU doctor places a PEG tube or the surgeon performs a gastrostomy	2.90 ± 0.10	90.00%
		ICU doctors and nutritionists discuss the type and target value (protein and energy) of the nutrient solution, discuss the supplementation of trace elements, and finalize the nutritional formula. The initial protein target: 1.2–2.0 g/kg/day; The initial caloric target: 15–25 kcal/kg/day	2.95 ± 0.07	95.00%
		ICU nurses implement enteral nutrition therapy: the initial speed is 20 mL/h, which is increased by 10 mL/h every 4–6 h until the target dose is reached. The target dose is determined by ICU doctors	2.95 ± 0.07	95.00%

### Part I: Multidisciplinary nutrition management process for critically ill patients

#### Round 1 (5 steps)

For the 16 criteria in part 1, the number of experts rating each as appropriate or feasible ranged from 15 to 18 out of 20 (75–90%). After a group discussion, the qualitative feedback suggestions were revised as follows: In Step 2-Standard 2, two experts proposed adjusting the “measuring the height and weight of patients” to “filling in the height and weight of patients” because it is difficult to measure the height and weight of critically ill patients in bed. In Step 2-Standard 5, one expert suggested adjusting the frequency of nutrition risk screening from once a week to once every 2 days. In Step 3-Standard 6, two experts suggested that the nutritionist should evaluate the need for enteral nutrition adjustments both independently and jointly with the ICU doctor. In Step 3-Standard 10, one expert proposed clarifying the radiologist’s role, suggesting that the phrase “positioning by the radiologist” be revised to clarify that the radiologist did not perform tube placement but rather radiographically verified the correct placement of the intestinal feeding tube via X-ray imaging. In Step3-Standard 12, two experts proposed clarifying that the need for radiologist involvement was to ensure correct positioning of the tube, and one expert suggested refining the formulation of nutrition target values. In Step 4, one expert proposed refining the time standard of this step, which is “48 h after admission and during hospitalization.” In Step 4-Standard 14, three nurses proposed that the assessment of nutritional support should not be conducted solely by ICU nurses, and recommended that ICU doctors and nurses complete the assessment jointly. Another expert suggested that the frequency of nutrition risk assessment should be adjusted from once a week to once every 2–3 days. In Step 5-Standard 16, an expert suggested that the formulation of the target feeding amount should be refined, including protein and calorie requirements.

#### Round 2 (5 steps)

Notably, for the 16 criteria in part I, the number of experts rating each as appropriate or feasible ranged from 17 to 20 (85–100%). In Step4-Standard 14, one expert suggested that the first ultrasonic monitoring of gastric retention should be conducted by the doctor.

### Part II: Enteral nutrition support process

#### Round 1

For each of the 15 criteria in this part, between 15 and 18 experts (75–95%) rated it as appropriate or feasible. Qualitative feedback: After the group discussion, the following modifications to the standard were suggested: In Standard 3, regarding the contraindications of enteral nutrition, one expert suggested revising “intestinal ischemia” to “intestinal ischemia or bleeding,” and five experts suggested enhancing the evaluation of gastrointestinal function classification and adding “AGI grade III or IV” as a contraindication. Standard 10: Regarding the placement of nasogastric tubes, the experts suggested that the method of gastrointestinal tube insertion should not be limited to placement under ultrasound guidance, and accordingly, “the placement of nasogastric tubes under ultrasound guidance by ICU nurses” should be revised to “the placement of nasogastric tubes under ultrasound guidance by ICU nurses and other methods.” Standard 14: Regarding nutrition plan formulation, two experts recommended that specific brand names should not be mentioned or specified when selecting disease-specific formulas for patients with special conditions. One expert pointed out that the formulation of nutrition target values should be refined, including protein and calorie requirements.

#### Round 2

For the 15 criteria in part I, the number of experts rating each as appropriate or feasible ranged from 17 to 20 (85–100%). The qualitative feedback from one expert was that “The patient’s nutrient solution starts from 20 mL/h and only increases by 10 mL/h in 4–6 h. Is the speed too slow?” In response to this point, the steering group members organized a discussion and temporarily decided against making any revisions to this standard and supported their decision with previously reported guidelines.

### Final process

**Figure 1:**
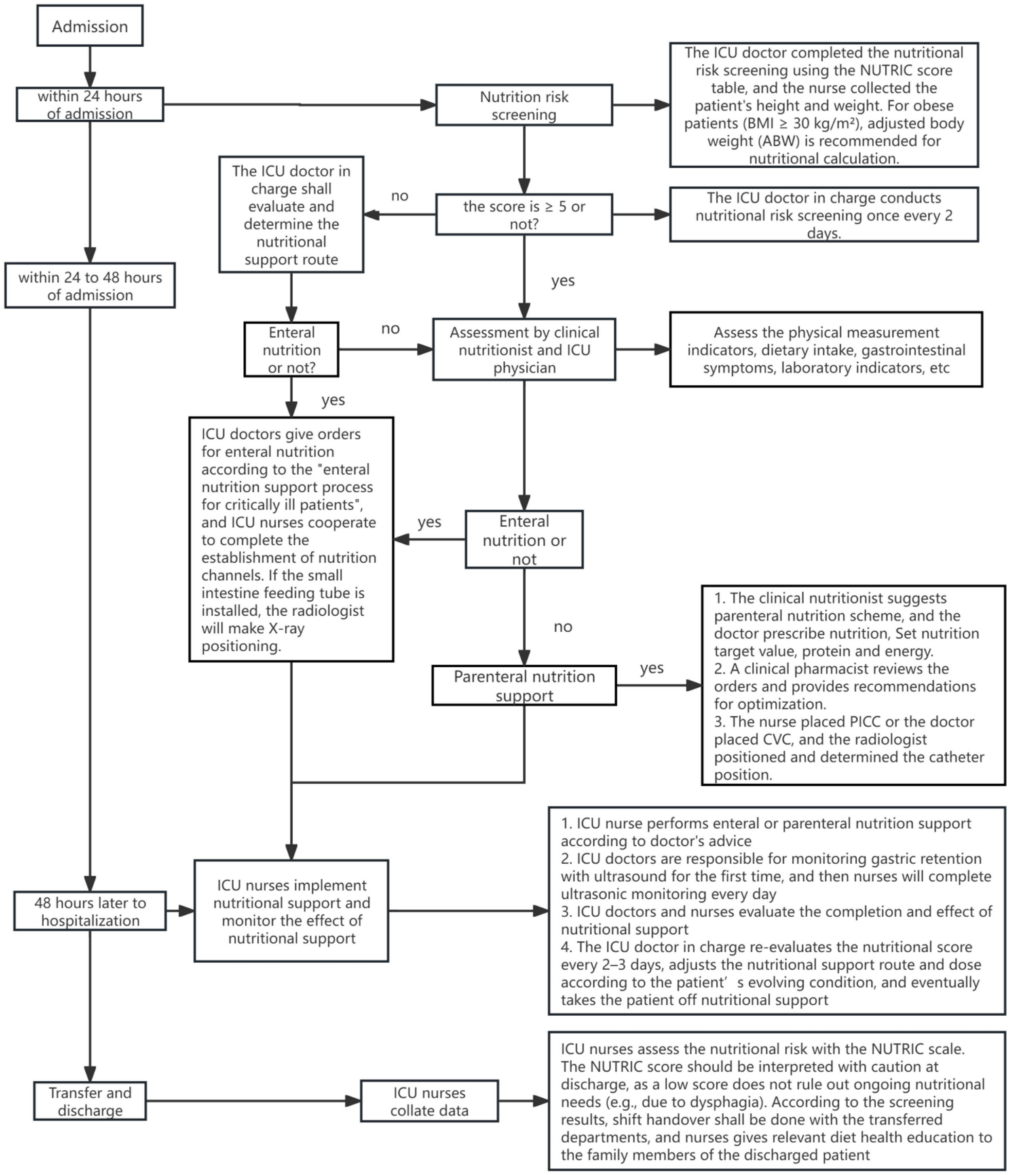
Standard process for nutrition management of multidisciplinary teams of critically ill patients.

**Figure 2:**
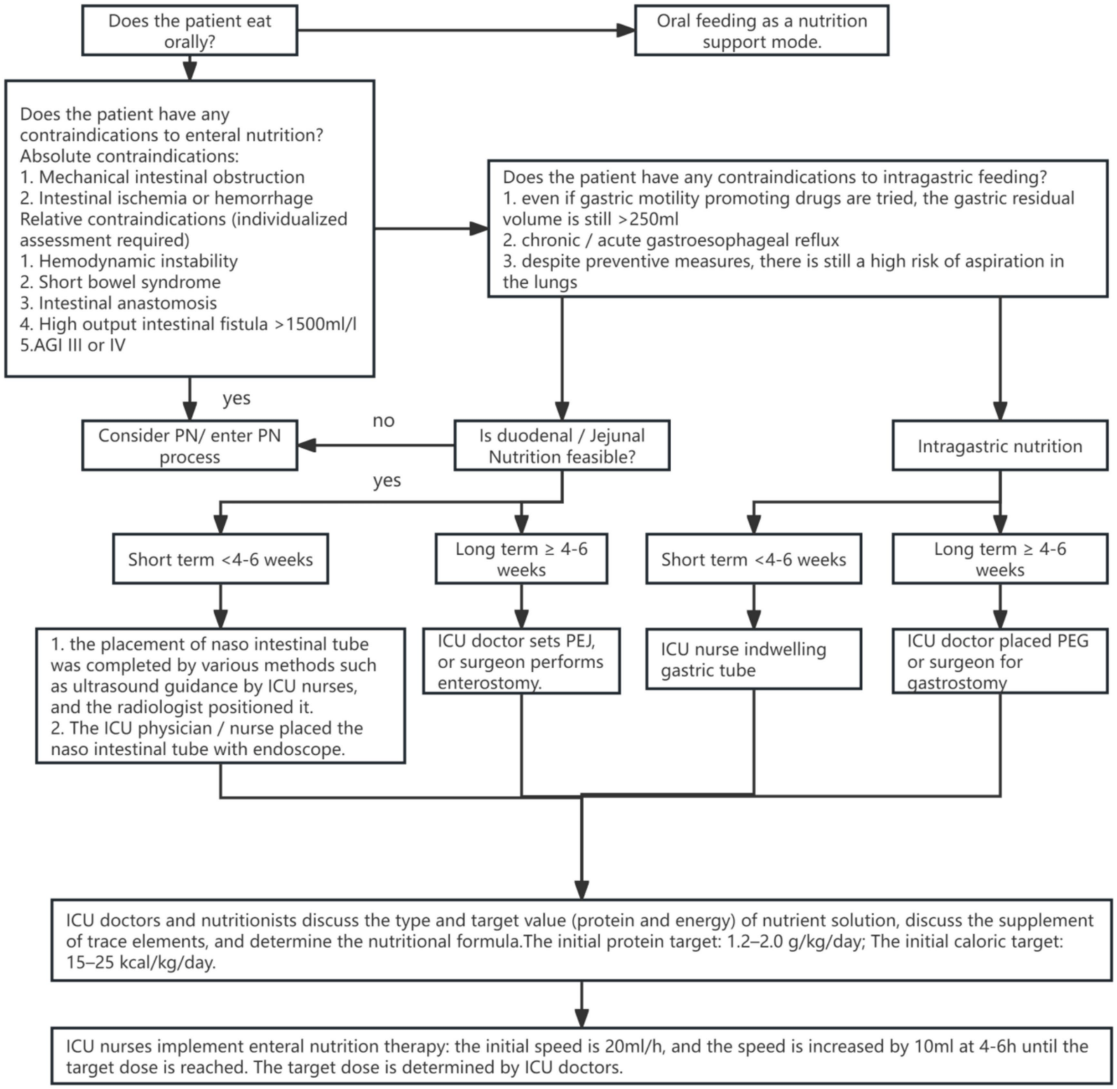
Standard process of enteral nutrition management of critically ill patients.

Overall, through screening and summarizing, the “multidisciplinary cooperative nutrition management process for critically ill patients” was finally formed. It included two parts, five steps, and 31 criteria. Our negligence at the beginning of implementing the Delphi method led to the omission of a standard—“the use of objective nutritional criteria to evaluate the gastrointestinal function and nutritional support effect of patients.” This omission was realized after the completion of Delphi’s second round of investigation. At the end of the Delphi method, all adjusted criteria were modified on the basis of the consensus of the steering group members. The steering group undertook an editorial process to summarize the evidence for external audiences. Figures 1 and 2 show the flow chart formed after the screening and generating the summary, respectively.

Nutritional targets and metabolic assessment: To ensure consistency in clinical application, the following explicit targets and methods are recommended based on the expert consensus and current guidelines. For critically ill patients, the initial protein target is 1.2–2.0 g/kg/day, and the initial caloric target is 15–25 kcal/kg/day, to be adjusted according to the phase of illness (acute vs. recovery). When available, indirect calorimetry is the preferred method for determining caloric needs; it should be performed by a trained clinical nutritionist or a respiratory therapist under the supervision of the ICU team. In the absence of indirect calorimetry, predictive equations (e.g., Penn State equation) may be used with caution. For obese patients (BMI ≥ 30 kg/m^2^), adjusted body weight (ABW) is recommended for nutritional calculation, where ABW = ideal body weight + 0.4 × (actual body weight – ideal body weight). Ideal body weight is calculated using the Devine formula (men: 50 kg + 2.3 kg per inch over 5 feet; women: 45.5 kg + 2.3 kg per inch over 5 feet). These specifications have been integrated into the multidisciplinary nutrition management process to reduce inter-provider variability.

## Discussion

### Principal findings

Through two rounds of expert consultation, we formed a multidisciplinary cooperative nutrition management process for critically ill ICU patients. The process was divided into two parts (“multidisciplinary cooperative nutrition management process for critically ill patients” and the “enteral nutrition support process”), five steps, and 31 standards. In the process of incorporating the feedback of the experts, 1 step and 12 standards were modified and adjusted. The consulting experts also provided suggestions on establishing a multidisciplinary management information system to automatically capture index data and improve work efficiency. In the final five steps, 31 criteria were considered as desirable or necessary by 85–100% of the experts.

### Comparison with international guidelines and previous models

The multidisciplinary cooperative nutrition management process developed in this study not only aligns with but also markedly extends existing international frameworks for critical care nutrition. Although guidelines from the ESPEN and ASPEN provide evidence-based targets for energy and protein requirements, they lack granular, role-specific operational workflows tailored to the resource constraints and organizational structures of Asian ICUs.

(1) Operational integration vs. consultative models

Previous systematic reviews, such as the one by Eriksen et al. (9), have validated the efficacy of NSTs in reducing complications. However, traditional NST models described in Western literature typically operate as consultative bodies—external experts visiting the ICU (30). In contrast, the process validated in our Delphi study integrates multidisciplinary function directly into the daily operational workflow of the ICU staff (e.g., Criteria 7: “ICU doctors give orders… nurses cooperate… radiologist confirms”). This “embedded” model mirrors the high-intensity staffing approaches described by Rinaldi et al. (15) in antimicrobial stewardship, wherein daily, bedside multidisciplinary rounds were found to be superior to on-demand consultations in terms of minimizing multidrug-resistant organism colonization. Our findings suggest that for nutrition management to be effective in the high-tempo environment of the ICU, it must be an intrinsic part of the daily patient care schedule rather than an on-demand external consultation.

(2) Phase-adapted precision vs. static initiation

The shift from “early full feeding” to “phase-adapted nutrition” is a critical advancement in recent literature. Stoppe et al. (1) and McKeever et al. (4) have highlighted that aggressive feeding during the acute catabolic phase may be harmful because of autophagy inhibition and endogenous glucose production. Although standard protocols often simply mandate feeding within 24–48 h, our consensus process refined this into distinct steps: physiological stabilization within 24 h of hospitalization and dietitian assessment within 24–48 h. This stepwise progression aligns with the concept of “metabolic readiness” proposed in recent precision nutrition reviews, ensuring that the initiation of nutrition considers the patient’s metabolic condition rather than following a rigid temporal schedule.

(3) Integration of point-of-care ultrasound

The explicit inclusion of ultrasound for gastric residual volume (GRV) monitoring (Criteria 14) is a unique feature of our consensus, distinguishing it from older nursing-led protocols. Traditional protocols heavily rely on gastric aspiration, a method increasingly questioned for its inaccuracy and poor correlation with aspiration pneumonia. Li et al. (25) recently developed an ultrasound-guided enteral nutrition (EN) protocol and demonstrated that objective, non-invasive monitoring reduces feeding interruptions. Formalizing the role of radiologists and ultrasound-trained ICU nurses in our process bridges the gap between outdated “blind” aspiration techniques and modern, visualizable precision medicine. This represents a significant upgrade from older Chinese ICU nursing routines and aligns with the global trend toward point-of-care ultrasound in critical care nutrition.

(4) Addressing the implementation gap in the Chinese context

Finally, literature specific to the Chinese healthcare settings has repeatedly identified a “guideline–practice gap” driven by high workload and role ambiguity (31). Yang noted that while Chinese intensivists recognize the importance of guidelines, guideline adherence is hampered by the lack of standardized, actionable workflows (31). By breaking the complex nutrition management guidelines into five chronological steps and 31 verifiable criteria, our study provides a practical “implementation scaffold.” This contradicts the broad, principle-based recommendations of national guidelines and offers a tangible tool that clarifies the roles and responsibilities of the involved personnel and the chronology of interventions, directly addressing the barriers of “lack of standardized training” identified in previous observational studies (32).

## Result in context

The findings of this study specified the guidelines of nutrition management of critically ill patients, which added operational clarity and facilitated multidisciplinary team cooperation. Nutrition management by a multidisciplinary team greatly benefits critically ill patients (9, 23); this has been confirmed in many studies (24) and is also recommended by nutrition guidelines (25, 33). However, a substantial gap remains between the guidelines and clinical application. Based on the available guidelines and existing research, a multidisciplinary cooperative nutrition management flow chart for critically ill patients was preliminarily developed in this study. Intervention measures in this flow chart had an assigned chronological sequence, and the responsibilities of ICU doctors, nurses, clinical nutritionists, clinical pharmacists, and radiologists on the nutrition management team were defined to standardize and facilitate the quality control of the nutrition management process. Based on elaborate consultations with experts, criteria of gastrointestinal function and nutritional status, and the nutrition target values were quantified at designated time points throughout the process, making the nutrition risk assessment and effect assessment more objective. Existing guidelines specify requirements regarding the frequency of nutritional risk assessment and the assessor’s minimum qualifications. During this consultation, the experts pointed out that such standards should be determined to improve the operability of the process. The use of the Delphi method and productive discussions within the research group can help establish a reliable, standardized, and convenient process for multidisciplinary nutrition management of critically ill patients.

### Implications of clinical practice and future health policy

The establishment of this standardized multidisciplinary nutrition management process can substantially improve the quality of clinical interventions, resource allocation, and patient safety in critical care settings.

### Enhancing clinical outcomes through protocolized precision

The primary implication of this protocol is the potential reduction in iatrogenic malnutrition and its associated sequelae. By mandating early screening with the mNUTRIC score—a tool specifically validated to identify patients who benefit most from aggressive nutritional intervention—and enforcing time-bound interventions, this process directly addresses the “delay to feed” phenomenon (7).

Furthermore, the explicit definition of caloric and protein targets, combined with the recommendation to use indirect calorimetry where feasible and a standardized method for weight calculation in obese patients, directly addresses the heterogeneity in nutritional management often observed across different ICUs. This standardization supports more accurate and equitable nutritional delivery.

### Optimizing economic and operational efficiency

Critically ill patients are resource-intensive, and malnutrition exacerbates this burden by prolonging mechanical ventilation and ICU length of stay. The explicit definition of roles in this protocol (e.g., pharmacist review of parenteral nutrition orders) is designed to minimize inappropriate prescribing and prevent metabolic complications. Eriksen et al. (9) reported that NSTs significantly reduced inappropriate parenteral nutrition use (by ~20%) and associated costs. Accordingly, implementing this protocol could yield substantial economic benefits for healthcare institutions by reducing waste and shortening high-cost ICU stays.

### Facilitating the integration of future technologies

This protocol lays the necessary groundwork for the integration of precision nutrition and artificial intelligence (AI). According to Stoppe et al. (1), the future of critical care lies in tailoring nutrient delivery to individual metabolic phenotypes. However, precision nutrition technologies cannot be effectively deployed in a disorganized environment. Standardizing the foundational workflow creates a structured data environment upon which advanced electronic clinical decision support systems (CDSS) can be layered. Future iterations could automate the calculation of energy targets and alert multidisciplinary team members about deviations, further reducing human error and enhancing compliance (23).

### Workforce development and interprofessional education

Finally, the clear delineation of responsibilities serves as a robust educational framework. It helps circumvent the obstacle of “role ambiguity” to effective teamwork (32). The protocol provides a curricular foundation for training ICU residents, specialized nurses, and clinical pharmacists, fostering a culture of collaboration that extends beyond nutrition to other domains of critical care management, such as sepsis control and trauma resuscitation.

### Future perspectives

The integration of electronic CDSS and AI represents a promising direction to enhance the precision and efficiency of the multidisciplinary nutrition management process proposed in this study. CDSS can automate risk screening, prompt time-bound assessments, and reduce documentation burden, and AI may facilitate dynamic, personalized nutrition adjustments through real-time analysis of physiological and biochemical data. Together, these technologies may improve clinical decision-making and protocol adherence and ultimately facilitate a shift toward truly patient-centered, precision nutrition care in critical settings. Future research should focus on co-designing and evaluating such systems in real-world ICU workflows to study their impact on both clinical outcomes and multidisciplinary collaboration.

## Strengths and limitations

This study used the Delphi method to construct a multidisciplinary cooperative nutrition management process for critically ill patients. Experts were selected from various regions of the country, facilitating good regional representation in the results. In the two rounds of consultation, experts showed remarkable interest and proactive involvement, and 70–90% of the 20 consultation experts suggested modifications. After elaborate discussions on the opinions of each expert, the research group revised the corresponding standards accordingly to incorporate the recommended revisions and make the items more representative. The final five steps and 31 standards are well recognized, and the degree of concordance among expert opinions was high. Additionally, the constructed protocol was preliminarily validated in one ICU ward in China, achieving favorable clinical outcomes in terms of early nutrition support rate and blood indicators such as serum albumin and prealbumin in critically ill patients.

This study has some limitations. First, the selected experts were all clinical medical staff and clinical nutritionists, with no clinical pharmacists or radiologists included, which may affect the representativeness of the findings. Second, as noted in the *Results*, an initial oversight led to the omission of a standard (*the use of objective nutritional criteria to evaluate the gastrointestinal function and nutritional support effect of patients*), which may have impacted the initial comprehensiveness of the process. Third, in the consultation questionnaire, the use of a 3-point Likert scale may have introduced ceiling effects. In future research, a 5-point Likert scale can be used to address this limitation. Finally, All experts were from China; therefore, applicability to other healthcare systems requires further validation.

Additionally, although indirect calorimetry is recommended as the gold standard for determining caloric needs, its availability remains limited in many Chinese ICUs. Therefore, most participating centers currently rely on predictive equations, which may introduce inaccuracies. Future implementation studies should evaluate the feasibility and impact of integrating indirect calorimetry into routine practice.

## Conclusion

Through two expert consultation rounds according to the Delphi method, a total of 20 ICU doctors, nurses, and clinical nutritionists from five provinces and cities in China participated in the consultation. They provided ratings and qualitative feedback on the nutrition management process, and accordingly, we developed a multidisciplinary nutrition management process for critically ill patients. The process is divided into two parts: a multidisciplinary nutrition management process for critically ill patients and an enteral nutrition support process, collectively including five steps and 31 standards. The newly developed process is strengthened by its foundation of reliable theoretical sources, objective evaluation criteria, and strong operability, thus supporting more comprehensive and personalized nutrition management of critical patients.

## Data Availability

The original contributions presented in the study are included in the article/Supplementary material, further inquiries can be directed to the corresponding author.

## References

[1] Stoppe C Hill A Christopher KB Kristof AS. Toward precision in nutrition therapy. Crit Care Med. (2025) 53:e429–40. doi: 10.1097/CCM.0000000000006537, 39688452 PMC11801434

[2] Mart MF Girard TD Thompson JL Whitten-Vile H Raman R Pandharipande PP . Nutritional risk at intensive care unit admission and outcomes in survivors of critical illness. Clin Nutr. (2021) 40:3868–74. doi: 10.1016/j.clnu.2021.05.005, 34130034 PMC8243837

[3] Xie M Huang L Li L Qin Y Feng B Cai Q . Association between NUTRIC score and ICU mortality in patients with sepsis: a prospective cohort study. Front Nutr. (2025) 12:1654901. doi: 10.3389/fnut.2025.1654901, 40823032 PMC12350113

[4] McKeever L Peterson SJ Lateef O Braunschweig C. The influence of timing in critical care nutrition. Annu Rev Nutr. (2021) 41:203–22. doi: 10.1146/annurev-nutr-111120-114108, 34143642

[5] Zeng QM Chen WF Zhang JH Han ZL Liao Y Zhang XQ . Multidisciplinary nutrition management practice for radiotherapy patients with head and neck tumors. J Nurs Sci. (2019) 34:97–101. doi: 10.3870/j.issn.1001-4152.2019.11.097

[6] Fukushima R Compher CW Correia MITD Gonzalez MC McKeever L Nakamura K . Recognizing malnutrition in adults with critical illness: guidance statements from the global leadership initiative on malnutrition. Clin Nutr. (2025) 49:202–8. doi: 10.1016/j.clnu.2025.03.011, 40169339

[7] Kim HJ Shim JC Oh JH Choi SB Lee HP Chang Y. A novel nutritional assessment tool combining the mNUTRIC score and the GLIM criteria with prognostic value for in-hospital mortality in critically ill patients: a single-center retrospective cohort study. Am J Clin Nutr. (2025) 122:306–14. doi: 10.1016/j.ajcnut.2025.05.005, 40354938

[8] Maekawa K Ohara N Higashibeppu N Kawamoto M Ohta T. Early nutritional support enhances recovery after endovascular thrombectomy: a prospective study with institutional historical control. Clin Nutr. (2025) 54:99–109. doi: 10.1016/j.clnu.2025.09.013, 41046780

[9] Eriksen MK Crooks B Baunwall SMD Rud CL Lal S Hvas CL. Systematic review with meta-analysis: effects of implementing a nutrition support team for in-hospital parenteral nutrition. Aliment Pharmacol Ther. (2021) 54:560–70. doi: 10.1111/apt.16530, 34275167 PMC9292190

[10] Merlino Barr S Fenton TR Hand RK Robinson DT Kim JH Groh-Wargo S. Perspective: improving neonatal registered dietitian nutritionist staffing, utilization, and compensation. Adv Nutr. (2025) 16:100417. doi: 10.1016/j.advnut.2025.100417, 40139314 PMC12056252

[11] Rosseel Z Overwater NMP Aerts M Chapple LAS Chen D Haines KL . Towards optimised nutrition therapy after critical illness: a position statement and research framework by the global research initiative on post-intensive care nutrition (GRIP) consortium. Crit Care. (2025) 29:460. doi: 10.1186/s13054-025-05710-2, 41163099 PMC12574275

[12] Moisey LL Merriweather JL Drover JW. The role of nutrition rehabilitation in the recovery of survivors of critical illness: underrecognized and underappreciated. Crit Care. (2022) 26:270. doi: 10.1186/s13054-022-04143-5, 36076215 PMC9461151

[13] Berger MM Burgos R Casaer MP de Robertis E Delgado JCL Fraipont V . Clinical nutrition issues in 2022: what is missing to trust supplemental parenteral nutrition (SPN) in ICU patients? Crit Care. (2022) 26:271. doi: 10.1186/s13054-022-04157-z, 36088342 PMC9464377

[14] Dickerson RN Andromalos L Brown JC Correia MITD Pritts W Ridley EJ . Obesity and critical care nutrition: current practice gaps and directions for future research. Crit Care. (2022) 26:283. doi: 10.1186/s13054-022-04148-0, 36127715 PMC9486775

[15] Rinaldi M Gatti M Tonetti T Nocera D Ambretti S Berlingeri A . Impact of a multidisciplinary management team on clinical outcome in ICU patients affected by gram-negative bloodstream infections: a pre-post quasi-experimental study. Ann Intensive Care. (2024) 14:36. doi: 10.1186/s13613-024-01271-9, 38448761 PMC10917714

[16] Bloom MW Vo JB Rodgers JE Ferrari AM Nohria A Deswal A . Cardio-oncology and heart failure: a scientific statement from the Heart Failure Society of America. J Card Fail. (2025) 31:415–55. doi: 10.1016/j.cardfail.2024.08.045, 39419165 PMC12317758

[17] Ke L Lin J Doig GS van Zanten ARH Wang Y Xing J . Actively implementing an evidence-based feeding guideline for critically ill patients (NEED): a multicenter, cluster-randomized, controlled trial. Crit Care. (2022) 26:46. doi: 10.1186/s13054-022-03921-5, 35172856 PMC8848648

[18] Chen X Beilman B Gibbs HD Hamilton JL Parker N Bur AM . Nutrition in head and neck cancer care: a roadmap and call for research. Lancet Oncol. (2025) 26:e300–10. doi: 10.1016/S1470-2045(25)00087-7, 40449504 PMC12915487

[19] Sharma K Mogensen KM Robinson MK. Pathophysiology of critical illness and role of nutrition. Nutr Clin Pract. (2019) 34:12–22. doi: 10.1002/ncp.10232, 30580456

[20] Singer P Blaser AR Berger MM Calder PC Casaer M Hiesmayr M . ESPEN practical and partially revised guideline: clinical nutrition in the intensive care unit. Clin Nutr. (2023) 42:1671–89. doi: 10.1016/j.clnu.2023.07.011, 37517372

[21] Oami T Chihade DB Coopersmith CM. The microbiome and nutrition in critical illness. Curr Opin Crit Care. (2019) 25:145–9. doi: 10.1097/MCC.0000000000000582, 30855323 PMC6499930

[22] Liu XX Li W Huo XP Yu K. A randomized controlled study on the effect of nursing led multidisciplinary cooperation on gastrointestinal function and nutritional status in elderly patients with colon cancer. Chin J of Clin Nutr. (2021) 29:135–41. doi: 10.3760/cma.j.cn115822-20210223-00041

[23] Stoian M Andone A Bândilă SR Onișor D Babă DF Niculescu R . Personalized nutrition strategies for patients in the intensive care unit: a narrative review on the future of critical care nutrition. Nutrients. (2025) 17:1659. doi: 10.3390/nu17101659, 40431399 PMC12114248

[24] Hayashi M Nishikido Y Banno H Michitaka T Tachibana E Tsukahara T. Effectiveness of registered dietitian-led management of early nutritional support in the emergency intensive care unit: a retrospective observational study. BMC Nutr. (2024) 10:96. doi: 10.1186/s40795-024-00904-3, 38970089 PMC11225280

[25] Li Z He W Tian D Sun Y Yang Q Cao L. Developing an ultrasound-guided enteral nutrition protocol for critically ill patients based on the Delphi method. Nurs Crit Care. (2025) 30:e70023. doi: 10.1111/nicc.70023, 40188845

[26] Lee SH Lee JG Kwon MK Kim J Kim M Park J . Nutritional support for critically ill patients by the Korean Society for Parenteral and Enteral Nutrition—part I: a clinical practice guideline. Ann Clin Nutr Metab. (2024) 16:89–111. doi: 10.15747/ACNM.2024.16.3.89

[27] Lepre B Mansfield KJ Ray S Beck EJ. Establishing consensus on nutrition competencies for medicine: a Delphi study. BMJ Nutr Prev Health. (2024) 7:68–77. doi: 10.1136/bmjnph-2023-000807, 38966103 PMC11221290

[28] Wei J Fang X Qiao J Liu H Cui H Wei Y . Construction on teaching quality evaluation indicator system of multi-disciplinary team (MDT) clinical nursing practice in China: a Delphi study. Nurse Educ Pract. (2022) 64:103452. doi: 10.1016/j.nepr.2022.103452, 36152471

[29] Hardman JC Harrington K Roques T Sood S Jose J Lester S . Methodology for the development of National Multidisciplinary Management Recommendations using a multi-stage meta-consensus initiative. BMC Med Res Methodol. (2022) 22:189. doi: 10.1186/s12874-022-01667-w, 35818027 PMC9275134

[30] Mistiaen P Van den Heede K. Nutrition support teams: a systematic review. JPEN J Parenter Enteral Nutr. (2020) 44:1004–20. doi: 10.1002/jpen.1811, 32181928

[31] Yang L Dong Z. Adherence to guidelines on nutritional support by medical residents in an intensive care unit in China: a prospective observational study. Med Sci Monit. (2019) 25:8645–50. doi: 10.12659/MSM.917684, 31733142 PMC6874836

[32] Thu E Voldsund S Sand K. Barriers to the delivery of enteral nutrition in intensive care unit patients – a descriptive cross-sectional study. Sykepleien Forskning. (2025) 20:e-98756. doi: 10.4220/Sykepleienf.2025.98756en, 41213920

[33] Girón K Chinchilla I Gómez C Lau M Oroxon MR Díaz E . Expert consensus on the nutrition care process in Guatemalan hospitals: findings from a Delphi study of nutrition day 2022 participants. Nutrients. (2025) 17:3110. doi: 10.3390/nu17193110, 41097187 PMC12525630

