# Supplementary material for: Development of a multidisciplinary cooperative nutrition management process for critically ill patients—a Delphi expert consensus study
Source: Front Nutr. 2026 Jun 19;13:1793038. doi: 10.3389/fnut.2026.1793038 (PMC13327916; doi:10.3389/fnut.2026.1793038)
Supplement: Supplementary file 1 [file Supplementary_file_1.docx]

Appendix 1: Expert consultation and modification comments on the "Multidisciplinary Enteral Nutrition Management Process for Critically Ill Patients".

| **Part** | **Step** | **Order** | Criteria | **Modification Comments** | **Modification Criteria** |
| --- | --- | --- | --- | --- | --- |
| Multidisciplinary nutrition management process for critically ill patients | admission | 1 | Nutrition risk screening |  |  |
|  | within 24 hours of admission | 2 | The ICU doctors use the NUTRIC score table to complete the nutritional risk screening, and the nurses measure the patient's height and weight | **Expert 4:** It is difficult to measure the weight of critically ill patients, and the difference is large;  **Expert 10:** "The nurse measures the patient's height and weight" is revised to "the nurse fills in the height, weight and weight";  **Expert 15:** You can contact IT to design s system for automatically capturing the scoring information and reduce the workload of studies. |  |
|  |  | 3 | the score is ≥ 5 or not? | **Expert 12:** If the score is greater than 5, it is recommended to evaluate whether the haemodynamics are stable, to determine whether to start nutritional support immediately. |  |
|  |  | 4 | The ICU doctor in charge shall evaluate and determine the nutritional support route |  |  |
|  |  | 5 | Continue weekly nutrition risk screening by the ICU doctor in charge | **Expert 4:** The condition of critically ill patients changes rapidly. It is recommended to evaluate it every day, at least once every two days. One week is too long. |  |
|  | within 24 to 48 hours of admission | 6 | Dietitian consultation assessment | **Expert 3:** It is appropriate for the nutritionist and ICU to assess this together;  **Expert 15:** Score ≥ 5, should be assessed by the nutritionists and ICU doctors together. |  |
|  |  | 7 | Enteral nutrition or not? |  |  |
|  |  | 8 | Assess the physical measurement indicators, dietary intake, gastrointestinal symptoms, laboratory indicators, etc. |  |  |
|  |  | 9 | Enteral nutrition or not? |  |  |
|  |  | 10 | The ICU doctors give orders for enteral nutrition according to the "flow chart of enteral nutrition support for critical patients", and the ICU nurses cooperate to complete the establishment of nutrition channels. Radiologist positioning. | **Expert 1:** "Radiologist positioning" is suggested to be revised to "if the small intestine feeding tube is installed, X-ray positioning is required". |  |
|  |  | 11 | Parenteral nutrition support |  |  |
|  |  | 12 | 1. the clinical nutritionist suggests the parenteral nutrition scheme, and the doctor gives nutrition doctor's advice.  2. the pharmacist checks the doctor's orders and puts forward professional opinions.  3. The nurse placed a PICC or the doctor places a CVC catheter. The radiologist determines the positioning. | **Expert 2:** "Radiologist positioning" is only recommended for a "PICC tube";  **Expert 9:** It is suggested to determine the nutrition target value: protein and calories;  **Expert 10:** It is suggested to modify that “the radiologist shall locate and confirm the position of the catheter”. |  |
|  | during hospitalization | 13 | The ICU nurses implement nutritional support and monitor the effect of nutritional support | **Expert 10:** It is suggested to adjust the expression of hospitalization period. The first 24 hours and 48 hours are included in the hospitalization period. |  |
|  |  | 14 | 1. The ICU nurses perform enteral or parenteral nutrition support according to the doctor's advice;  2. The ICU nurses use ultrasound to monitor the amount of gastric retention;  3. The ICU nurses observed the completion and effect of nutritional support;  4. The ICU doctor in charge shall re-evaluate the NUTRIC score every week, adjust the nutritional support route and dose according to the changes in the patient's condition and the completion of nutritional support, and ask the clinical dietitian for consultation if necessary. | **Expert 1:** It is suggested that the "ICU nurse" should be changed to the "ICU doctor and nurse".  **Expert 4:** The effect of nutritional support should be assessed by the ICU doctor and nurse; The doctor should evaluate NUTRIC every 2-3 days.  **Expert 5:** Is the first measurement made by a doctor or a nurse?  **Expert 10:** It is suggested that the daily monitoring of gastric retention by ultrasound should be completed by the doctor and nurse.  **Expert 12:** It is recommended to use the enteral nutrition tolerance score.  **Expert 14:** the significance of the daily monitoring of gastric retention is not as great as that of clinical symptoms; It is recommended to evaluate the tolerance of enteral nutrition every 4-6 hours (or nurse shift) after the start of enteral nutrition; If abdominal pain, nausea and vomiting happen, report to the doctor for a decision: reduce, maintain the original amount, or increase the amount of feeding. |  |
|  | transfer and discharge | 15 | Nurses collect relevant data | **Expert 10:** It is suggested to revise to the "nurses sort out the data". |  |
|  |  | 16 | 1. The ICU collects relevant data of the patients: general data, mechanical ventilation days, ICU hospitalization days, total hospitalization days, total hospitalization expenses, antibiotic expenses, disease outcome, etc.;  2. When the patient is transferred out or discharged from the hospital, the ICU nurse again uses the NUTRIC scoring table to evaluate the nutritional risk, completes the shift handover with the transferred department according to the screening results, and the discharged patient gives relevant diet health education to the family members. | **Expert 12:** It is suggested to add direct indicators to evaluate the effect of nutritional support, such as the Completion of target feeding amount, weight, protein, metabolism, etc.；  **Expert 14:** It is suggested to add the indicator "days of antibiotic treatment". |  |
| Enteral nutrition support process for critically ill patients |  | 1 | Can the patient eat by mouth? |  |  |
|  |  | 2 | Oral feeding |  |  |
|  |  | 3 | Does the patient have any contraindications to enteral nutrition?  Absolute contraindications:  1. mechanical intestinal obstruction;  2. intestinal ischaemia.  Relative contraindications (individualized assessment required)  1. haemodynamic instability;  2. short bowel syndrome;  3. intestinal anastomosis;  4. high output intestinal fistula >1500 ml/d | **Expert 5:** It is suggested to add contraindications: gastrointestinal bleeding;  **Expert 6:** Are the AGI grades supplemented for contraindications?  **Expert 9:** Use the AGI classification to evaluate the gastrointestinal function;  **Expert 12:** It is recommended to determine whether to use enteral nutrition through the gastrointestinal function (AGI) classification;  **Expert 13:** It is suggested to determine whether to use enteral nutrition or other more quantifiable evaluation indicators through the gastrointestinal function (AGI) classification.  **Expert 14:** It is recommended to include the intestinal function classification (AGI) in the assessment; It is recommended to first assess if there is a high nutritional risk or if the patient cannot take food by mouth for more than three days. Assess the gastrointestinal function with the AGI grade. |  |
|  |  | 4 | Does the patient have any contraindications to intragastric feeding?  1. even if gastric motility promoting drugs were used, the gastric residual volume was still >250 ml;  2. chronic/acute gastroesophageal reflux;  3. despite the use of preventive measures, there is still a high risk of lung aspiration. | **Expert 18:** High risk of aspiration: feeding through naso intestinal tube; Low risk of aspiration: feeding through gastric tube. |  |
|  |  | 5 | Consider pn/enter PN process |  |  |
|  |  | 6 | Is duodenal/Jejunal Nutrition feasible? | **Expert 14:** Is duodenal/Jejunal Nutrition feasible here? Was it clear or excluded when assessing whether enteral nutrition could be given? |  |
|  |  | 7 | Intragastric nutrition |  |  |
|  |  | 8 | Short term <4-6 weeks |  |  |
|  |  | 9 | Long term >4-6 weeks | **Expert 10:** Is it necessary to make a fistula after 4-6 weeks? |  |
|  |  | 10 | 1. The nurse in the ICU will perform the placement of a naso intestinal tube under the guidance of ultrasound; The radiologist will confirm the positioning.  2. The doctors/nurses in ICU can place a naso intestinal tube under endoscopy. | **Expert 16:** It is suggested that the placement should be changed to indwelling. In addition to ultrasound guidance, nurses have other methods to place naso intestinal tubes. |  |
|  |  | 11 | The ICU doctor in charge places a PEJ, or the surgeon performs an enterostomy. | **Expert 10:** PEG\PEJ is also a method of fistula. Surgeons can also choose this method for fistula. The description needs to be differentiated. |  |
|  |  | 12 | The ICU nurse places a gastric tube. | **Expert 17:** It is suggested to change resettlement into retention.. |  |
|  |  | 13 | The ICU doctor in charge places a peg or the surgeon performs a gastrostomy | **Expert 12:** Is PEG or PEJ a routine treatment for long-term enteral nutrition support? |  |
|  |  | 14 | The ICU doctors have discussions with the clinical nutritionists to determine the type of nutrient solution: diabetes patients should have ruidai, the patients with high calorie demand but limited fluid intake should have Ruixian, and the patients with low immunity should have jiaweiti or nutrition preparations. | **Expert 9:** Should the nutrition target value be determined with protein and heat card; the supplement of trace elements and the formula should be considered.  **Expert 4:** Patients with special diseases should have the special disease formula without specifying the manufacturer.  **Expert 10:** Is it necessary to write down the trade name? There are some similar products. |  |
|  |  | 15 | Enteral nutrition therapy in the ICU: the initial speed is 20 ml/h, and the speed is increased by 10 ml every 4-6 h until the target dose is reached. The target dose is determined by the ICU doctors. | **Expert 14:** It is recommended to evaluate the tolerance of enteral nutrition (tolerance score/GVR/clinical symptoms) every 4-6 hours to adjust the nutritional dosage and feeding amount, and whether the actual calorie intake value reaches 60% of the target amount after 7-10 days; If it is reached, continue to gradually increase to the feeding amount. If it is not reached, it is recommended to add intravenous nutrition.  **Expert 10:** Every patient starts from 20 and increases by 10 after 4-6 hours. This initial speed is a little slow. |  |
